# Elevated fasting insulin predicts the future incidence of metabolic syndrome: a 5-year follow-up study

**DOI:** 10.1186/1475-2840-10-108

**Published:** 2011-11-30

**Authors:** Ki-Chul C Sung, Mi-Hae H Seo, Eun-Jung J Rhee, Andrew M Wilson

**Affiliations:** 1 Division of Cardiology, Department of Internal Medicine, Kangbuk Samsung Hospital, Sungkyunkwan University School of Medicine, Seoul, South Korea; 2 Division of Endocrinology and Metabolism, Department of Internal Medicine, Kangbuk Samsung Hospital, Sungkyunkwan University School of Medicine, Seoul, South Korea; 3 Department of Medicine, University of Melbourne, St. Vincent's Hospital, Melbourne, Australia

**Keywords:** Metabolic syndrome, hyperinsulinemia, insulin Resistance

## Abstract

**Background:**

There is controversy about the specific pathophysiology of metabolic syndrome (MS) but several authors have argued that hyperinsulinemia is a key feature of the cluster. We aimed to assess whether the baseline insulin levels could predict the development of MS in a well characterised cohort of otherwise healthy adults who were followed over a five year period.

**Methods:**

We identified 2, 350 Koreans subjects who did not have MS in 2003 and who were followed up in 2008. The subjects were divided into 4 groups according to the baseline quartiles of fasting insulin, and the predictors of the incidence of MS were analyzed using multivariate regression analysis.

**Results:**

Over the follow up period, 8.5% of the cohort developed MS. However, 16.4% of the subjects in the highest quartile of the insulin levels developed MS. In a model that included gender, age, the smoking status, the exercise level, alcohol consumption and the systolic blood pressure, the subjects in the highest quartile of the insulin levels had more than a 5 times greater risk of developing MS compared that of the subjects in the lowest quartile. This predictive importance remained significant even after correcting for all the individual features of MS.

**Conclusions:**

These data suggest that high baseline fasting insulin levels are independent determinants for the future development of MS.

## Background

Metabolic syndrome (MS) has received increased attention as an important epidemiological tool for predicting cardiovascular disease (CVD) and type 2 diabetes (T2DM) [1-8]. MS is a cluster of CVD and T2DM risk factors with a shared pathophysiology [2,5,9,10]. Although there is still debate about insulin resistance and hyperinsulinemia being central to the clustering of these common CVD risk factors [11,13], they have been recognized as pathophysiologic linking factors of MS [14-16]. Early and identification of those people who are most likely to develop the MS and/or its component features should lead to targeted and "tailored" intervention strategies, and particularly lifestyle modifications such as exercise and dietary changes [17].

So far, there have been only limited data of the relationship between the future development of MS and the baseline insulin level, which is a surrogate maker of insulin sensitivity/resistance. Thus, in this large, well characterized, cohort study of otherwise healthy subjects without T2DM or MS, we aimed to assess whether the baseline insulin levels can predict the development of MS. We hypothesized that measuring the fasting insulin in healthy subjects can predict the subsequent development of MS.

## Methods

**Figure 1:**
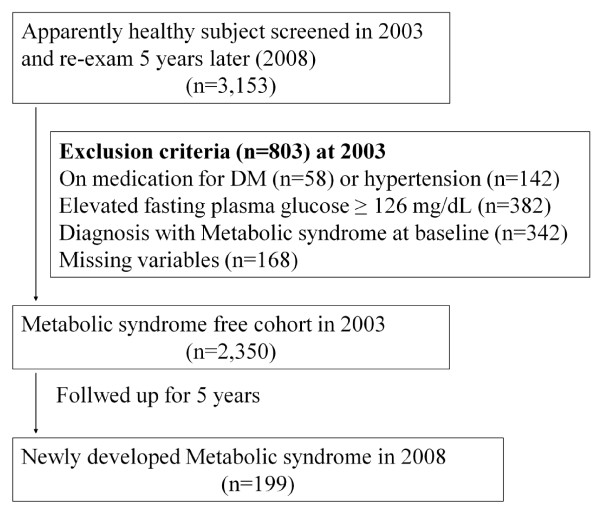
**Selection of study population**.

The study population consisted of apparently healthy Koreans who had a comprehensive health examination in 2003 and they were re-examined 5 years later (2008) at Kangbuk Samsung Hospital, College of Medicine, Sungkyunkwan University. Initially 3, 153 individuals were identified. Among these participants, 803 were excluded. Individuals were excluded if they were on medication for T2DM (n = 58) or hypertension (n = 142) and/or if they had an elevated fasting plasma glucose level ≥ 126 mg/dL (n = 382). Three hundred forty two participants were excluded for being diagnosed with MS at baseline. Individuals were also excluded for absence of data including fasting insulin level (n = 168). Thus, 2, 350 participants were eligible and included for the study (Figure 1).

The study was approved by the institutional review board at Kangbuk Samsung Hospital. The health examination consisted of a full medical history and comprehensive blood test evaluation. The participants' height and weight were measured barefoot and in light clothing. The body mass index (BMI) was calculated as weight in kilograms divided by the height in square meters. Waist circumference was measured with the subject in the standing position and at the level of umbilicus by a single examiner. Blood pressure was measured using a standardized sphygmomanometer after 5 minutes of rest. Questionnaires were used to ascertain information regarding the frequency of exercise (none, < 1 time/week, ≥1 time/week), the smoking status (never, past, current) and the amount of alcohol consumed (both quantity and frequency). Current smoker were define as those who admitted to smoking at the interview. Grams of alcohol consumption were calculated by multiplying the frequency by the amount, as previously reported [18].

Laboratory examinations were obtained after an overnight fast. The fasting plasma glucose, total cholesterol (TC), triglyceride(TG), low-density lipoprotein cholesterol (LDL-C) and high density lipoprotein cholesterol (HDL-C) concentrations were measured using Bayer Reagent Packs on an automated chemistry analyzer (Advia 1650 Autoanalyzer; Bayer Diagnostics, Leverkusen, Germany) [18,19]. High sensitivity CRP was measured by immunonephelometry (Nephelometry, Behring Nephelometer II, Dade Behring Marburg GmbH, Germany) with a detection limit of 0.2 mg/L (the values < 0.2 mg/L were regarded to be 0.1 mg/L). The fasting insulin concentration as a surrogate measurement of insulin sensitivity was measured with an immunoradiometric assay (Biosource, Nivelle, Belgium) with intra- and interassay coefficients of variation of 2.1- 4.5% and 4.7-12.2%, respectively.

Patients were classified as having MS if he or she had three or more of the following criteria: waist circumference ≥ 90 cm in men or ≥ 80 cm in women, elevated triglycerides ≥150 mg/dl, reduced HDL cholesterol < 40 mg/dl in men and < 50 mg/dl in women, blood pressure: ≥130/85 mmHg or treatment of previously diagnosed hypertension, elevated fasting glucose ≥ 100 mg/dl or treatment of previously diagnosed T2DM [2]. Patient was diagnosed as having diabetes mellitus after 5 years, if she or he was being treated for diabetes or if the fasting glucose level was higher than or same as 126 mg/dL according to the criteria from American Diabetes Association [20].

The continuous variables were reported as mean values with standard deviations and they were compared using the independent t-test. Categorical variables were expressed as percentages and they were compared using χ^2^-tests. The relationship between the baseline insulin level and the future development of MS was analysed in 2 steps. First, the subjects were divided into 4 groups according to the quartiles of the baseline insulin level (IU/mL): I (≤6.01), II (6.02-7.29), III (7.30-8.97) and IV (≥8.98). We then used logistic regression analysis to determine the odds ratio (OR) of developing metabolic syndrome in the individuals stratified by the quartiles of the fasting insulin concentration (which was used as a surrogate measure of insulin resistance). Second, to better understand the role of hyperinsulinemia for increasing the risk of MS and this was independent of any MS component at baseline, we excluded the subjects who had any MS component at baseline and we reanalysed the data with logistic regression analysis. We conducted both unadjusted and adjusted analyses. Adjustments were made for the following variables: gender, age, the smoking status, the exercise level (less than once a week or at least once a week), the amount of alcohol the subject drank (grams/day) and the metabolic parameters (systolic blood pressure, HDL-Cholesterol, triglyceride, fasting glucose, waist circumference). All the significance tests were 2-tailed, and *p *values < 0.05 were considered to be statistically significant. All the statistical analyses were performed using SPSS version 17.0 software (SPSS Inc., Chicago, IL, USA).

## Results

During the mean follow up of 5 years, 199 of 2350 subjects (8.5%) developed MS. Table 1 summarizes the baseline characteristics of the study participants according to the quartiles of baseline fasting insulin levels. There were significant differences in the demographics and risk factors between as the fasting insulin increases from first quartile to 4^th ^quartile. As the baseline fasting insulin level increased, the subjects were more likely to be obese and on average showed gradually increasing mean values for fasting glucose, fasting insulin, systolic blood pressure and lipid profiles.

**Table 1:** Baseline characteristics of the participants according to the insulin stratification

	I(≤6.01) (n = 592)	II (6.02-7.29) (n = 585)	III(7.30-8.97) (n = 588)	IV(≥8.98) (n = 585)	p-value
Age(yr) Male %	41.9 ± 6.3 75.5	42.0 ± 6.4 74.2	40.7 ± 6.2 73.8	40.7 ± 6.0 74.2	< 0.001 0.915
Life style					
Alcohol consumption (g/day)	12.6 ± 14.8	12.4 ± 16.9	11.3 ± 15.5	11.8 ± 14.8	0.206
Smoking % (No/Ex/Current)	47.1/18.6/34.3	51.3/20.3/28.4	49.0/23/28.1	42.6/26.8/30.6	0.003
Exercise % (none/less than once per week/more than once per week)	21.6/31.6/46.8	22.9/33.5/43.6	24.1/39.6/36.2	25/42.7/32.3	< 0.001
BMI(kg/m^2^)	22.7 ± 2.4	23.1 ± 2.5	23.8 ± 2.5	24.8 ± 2.8	0.018
Waist circumference (cm)	78.4 ± 8.2	79.1 ± 8.1	80.9 ± 8.1	83.7 ± 8.8	< 0.001
Systolic BP (mmHg)	112.9 ± 12.6	113.4 ± 12.4	114.5 ± 13.8	115.6 ± 13.6	< 0.001
Diastolic BP mmHg)	73.2 ± 9.6	73.4 ± 9.6	73.8 ± 10.1	74.5 ± 10.1	0.022
Fasting glucose (mg/dl)	87.4 ± 8.0	89.0 ± 7.9	90.9 ± 8.1	91.6 ± 7.6	< 0.001
Triglyceride (mg/dl)	112.4 ± 61.2	121.0 ± 64.3	135.2 ± 70.3	154.2 ± 82.6	< 0.001
HDL-cholesterol(mg/dl)	58.7 ± 11.8	58.2 ± 11.6	56.8 ± 11.6	55.3 ± 11.2	< 0.001
LDL-cholesterol(mg/dl)	117.2 ± 30.0	118.6 ± 30.0	119.6 ± 29.4	124.3 ± 31.0	< 0.001
hs-CRP(mg/L)	0.82 ± 1.16	0.78 ± 1.18	0.92 ± 1.46	1.15 ± 1.87	0.04

Values expressed as n (%) or mean ± standard deviation (by Student *t *-test)

BMI, body mass index; BP, blood pressure; hs-CRP, high-sensitivity C-reactive protein; MS, metabolic syndrome; DM, diabetes mellitus

**Figure 2:**
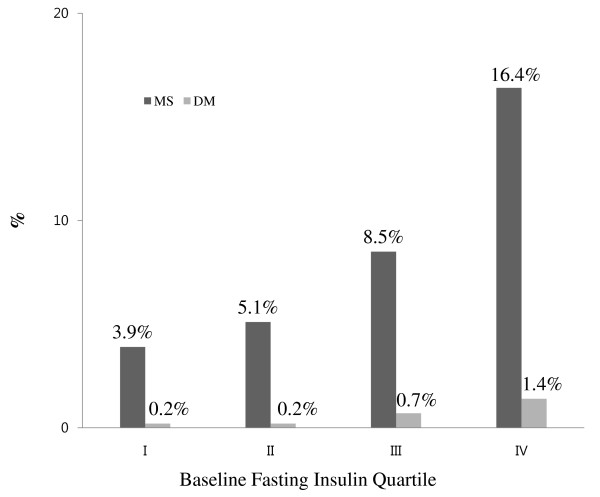
**Incidence MS and DM (%) according to baseline fasting insulin quartile in the whole cohort without any MS component at baseline**. Cut off points for the quartiles (IU/mL) were I: fasting insulin≤6.01, II: 6.02≤ fasting insulin ≤ 7.29, III: 7.30≤fasting insulin≤8.97, IV: fasting insulin≥8.98. MS, Metabolic syndrome; DM, diabetes mellitus.

As seen in Figure 2, the proportion of subjects who developed MS increased as the quartile of the baseline fasting insulin level increased from the first to the fourth quartile in the whole cohort of subjects without any MS component. For the subjects in the highest quartile for insulin levels, 16.4% subsequently developed MS in the whole cohort. The proportion of subjects who developed diabetes mellitus significantly increased as the baseline fasting insulin level increased from first to fourth quartile (Figure 2).

In the whole study cohort, hyperinsulinemia was an important predictor of future MS (Table 2). When the odd ratios for the development of MS, according to the quartile groups of the baseline fasting insulin level, were analysed with logistic regression analyses, the risk for the development of MS increased as the quartile of the baseline fasting insulin level increased from the first to the fourth quartile after correction for confounding variables.

**Table 2:** Incidence and odds ratios for the development of MS according to baseline insulin quartile in 5 years of follow-up in whole cohort

	I(≤6.01) (n = 592)	II (6.02-7.29) (n = 585)	III(7.30-8.97) (n = 588)	IV(≥8.98) (n = 585)
Incident MS %(n)	3.9 (23/592)	5.1% (30/585)	8.5% (50/588)	16.4% (96/585)

Odds ratio (95% CI)				

Model 1	1	1.4 (0.8 - 2.4)	2.5 (1.5 - 4.1) *	5.1 (3.1 - 8.2)*
Model 2	1	1.3 (0.7 - 2.4)	2.0 (1.2 - 3.4) †	3.6 (2.2 - 5.8)*
Model 3	1	1.2 (0.7 - 2.1)	1.7 (1.0 - 2.9)	3.0 (1.8 - 5.0)*
Model 4	1	1.1(0.6 - 2.1)	1.5 (0.9 - 2.5)	2.0 (1.2 - 3.3)†

Adjusted factors in the models:

Model 1: Gender, age, smoking status, exercise level, alcohol drinking amount, systolic blood pressure

Model 2: Model 1 + HDL-cholesterol, triglyceride

Model 3: Model 1 + HDL-cholesterol, triglyceride, fasting glucose

Model 4: Model 1 + HDL-cholesterol, triglyceride, fasting glucose, waist circumference

* *p *< 0.001

† *p *< 0.05

MS, metabolic syndrome; HDL, high-density lipoprotein; CI, confidence interval

After adjustment for gender, age, the smoking status, the exercise level, the amount of alcohol drank and the systolic blood pressure, the subjects in the highest quartile of the insulin levels had over a 5 times greater risk of developing MS as compared to that of the subjects in the lowest quartile (OR: 5.1, 95% CI: 3.1 - 8.2). This predictive importance remained significant even after adjustment for all the individual features of MS, and this underlines the potential importance of an elevated insulin level for predicting the development of MS (Table 2).

To better understand the role of hyperinsulinemia for increasing the MS risk independent of any MS component at baseline, we excluded the subjects who had any MS component at baseline (Table 3). Interestingly, in the previously healthy subjects who were without any MS component at baseline, elevated insulin was still a significant predictor of a future diagnosis of MS. The subjects in the highest quartile of the insulin levels had over a 10 times greater risk of developing MS compared to that of the subjects in the lowest quartile after adjustment for age, gender, the smoking status, the exercise level and the amount of alcohol drank (OR: 10.7, 95% CI: 2.4-47.9). However, Quartile 3 (OR: 2.3, 95% CI: 0.4-12.9) did not show a significantly increased risk for the development of MS compared with that of Quartile 2 (OR: 3.0, 95% CI: 0.6-15.2). Thus, there appeared to be a threshold effect in terms of the development of MS in that the incidence was only increased in the highest quartile and there was not a significant difference between quartiles 1, 2 and 3.

**Table 3:** Odds ratios for the development of MS according to baseline insulin quartile in 5 years of follow-up in subjects without any MS components at baseline

Quartiles of baseline fasting insulin level (IU/mL)	Incident MS	Odds ratio (95% CI)	
	
	n (%)	Unadjusted	**Adjusted**^**a**^
All	28/1171(2.4%)		
I(≤5.76)	2/289 (0.7%)	1	1
II(5.77-6.73)	6/294 (2.0%)	3.0(0.6-14.9)	3.0(0.6-15.2)
III(6.74-8.22)	4/292 (1.4%)	2.0(0.4-11.0)	2.3(0.4-12.9)
IV(≥8.23)	16/296 (5.4%)	8.2(1.9-36.0) *	10.7(2.4-47.9) *

Values expressed as n(%) or odds ratio (95% CI)

^a^Adjusted for age, gender, smoking status(never or past, current), exercise level(< 1 time/week, ≥1 time/week), alcohol drinking amount(in grams per day)

* p < 0.001

MS, metabolic syndrome; CI, confidence interval

For the whole cohort, subjects with high TG showed the highest incidence amongst 5 components of MS after 5 years (Table 4). The subjects without any MS components at baseline, hyperglycemia was the mostly developed components amongst 5 MS components after 5 years (Table 4).

**Table 4:** The development of individual MS components after 5 years

	Abdominal obesity	Hyperglycemia	Hypertension	Low HDL-C	High TG
Whole cohort	317/2350 (13.5%)	578/2350 (24.6%)	489/2350 (20.8%)	228/2350 (9.7%)	673/2350 (28.6%)
Subjects without any MS components at baseline	77/1171 (6.6%)	175/1171 (14.9%)	125/1171 (10.7%)	77/1171 (6.6%)	155/1171 (13.2%)

MS, metabolic syndrome; HDL-C, high density lipoprotein cholesterol; TG, triglyceride

## Discussion

We addressed the hypothesis that measuring the fasting insulin in healthy subjects would enable predicting the subsequent development of MS. This study has shown that in a general cohort of healthy Asian subjects, 8.5% developed MS over a 5 year period and that elevated fasting insulin levels predicted the development of subsequent MS even in the subgroup of patients without any MS component at baseline. This was particularly seen for the subjects in the highest quartile of the insulin levels when compared to those subjects with lower fasting insulin levels. Therefore, high baseline fasting insulin levels independently predicted the development of MS over time.

Numerous studies have been performed that showed that insulin resistance is associated with MS per se or the factors that comprise MS (e.g., NAFLD [21,22], T2DM [23,24], CVD [25]). In the study by Mykkänen et al [26]., insulin sensitivity assessed by frequently sampled intravenous glucose tolerance test and the minimal model showed significant association with the number of metabolic disorders. In addition, fasting hyperinsulinemia has been used as a surrogate estimate of insulin resistance according to various combinations of fasting insulin and the glucose concentration such as HOMA (homeostatic model assessment) [27,28], but not the fasting insulin level *per se*. Assuming that insulin resistance and the obesity being the central pathogenic factor in the development of MS, a recent study reported the role of leptin, an adipokine associated with obesity, on the prediction of MS and CVD risk [29]. However, our study is the only study that has measured the baseline insulin concentration as a surrogate marker in order to evaluate the risk of developing MS among individuals with and without any MS component and as stratified by the fasting insulin concentration.

Morrison et al. have recently shown that an interaction between the BMI and the HOMA score is associated with the development of MS in children [30] and De Boer et al. has shown in children that the features of MS are correlated with the fasting insulin levels [31]. Not surprisingly, higher fasting glucose within the normal range in children is associated with increased insulin levels, yet that study was correlative rather than prognostic [31]. Our study adds to the growing evidence by showing that elevated insulin occurs early and it predicts the subsequent development of MS. We have also found that an elevated fasting insulin level itself is potentially a key early feature in the pathophysiology of the clinical risk factor cluster of MS.

There have been many studies that have shown that patients with MS are at an increased risk of cardiovascular atherosclerosis [32-35]. Mehta et al. have recently shown that measures of insulin resistance, such as HOMA-IR, add predictive value over and above the diagnosis of MS itself in terms of the correlation with coronary artery calcification, which is a marker of the atherosclerotic risk [15]. In the study by Tenenbaum et al [36]., insulin resistance assessed by HOMA-IR was an independent predictor for new major cardiovascular events among patients with preexisting coronary artery disease. Therefore, strategies that screen patients' insulin levels and therapies that specifically target hyperinsulinemia may have value to prevent this common risk factor for CVD.

There are several limitations in our study. There is ongoing controversy about whether the concept of metabolic syndrome is useful to predict CVD and T2DM [2,5,9]. The diagnosis of MS has recently been shown to be useful for predicting subsequent CVD events, including in Asian cohorts [37] and in a recent meta-analysis [38]. Insulin sensitivity does not account for 100% of the variation in insulin response [13]. Kim et al. showed that the fasting insulin concentration reflects less than 40% of the variability in insulin resistance as measured by a direct technique, although the fasting insulin concentration is significantly associated with insulin resistance [28]. This study population was extremely well characterized, yet it was a homogenous population and it is representative of an adult Korean working population. This limits the generalizability of the data to other patient populations to some extent. Although the euglycemic insulin clamp test is currently the best and most accurate technique for assessing insulin sensitivity, the fasting insulin level was used as the marker for insulin resistance in this current study [39]. The reason we didn't use the euglycemic insulin clamp technique is that it is almost impossible to perform this test in all the participants in this kind of large study population. Lastly, we did not analyze the risk for MS with Cox proportional hazard model or Kaplan-Meir survival estimate. This study was done in subjects who performed health examination twice in 5 years of interval, 2003 and 2008. We cannot specify the exact point when the subjects developed the disease of interest during those 5 years. Therefore, Cox proportional hazard model or Kaplan-Meir survival estimate could not be used, and this could have lowered the significance of the results. However, our study result has its own meaning study in that high baseline insulin concentration predicts the risk of developing MS among individuals with and without any MS component and as stratified by the fasting insulin concentration.

In conclusion, this study is the first to evaluate elevated fasting insulin as an independent predictive factor for the development of subsequent MS over a 5 year period in a well characterized cohort of apparently healthy adults. This study supports the concept that hyperinsulinemia is an early feature, if not the central feature, of the cardiovascular clusters of MS. We have to keep in mind the importance of high fasting insulin levels not just as a surrogate marker to predict future development of MS, but also the increased risk for future CVD itself as well. The usefulness of strategies to reverse hyperinsulinemia, such as lifestyle modifications, on the prevention of MS and CVD, warrants further investigation.

## Competing interests

The authors declare that they have no competing interests.

## Authors' contributions

KS designed the study, analyzed the data and wrote the manuscript; MS and ER wrote and revised the manuscript; AMW wrote and edited the manuscript. All authors read and approved the final manuscript.
